# The CXCL12/CXCR4/ACKR3 Signaling Axis Regulates PKM2 and Glycolysis

**DOI:** 10.3390/cells11111775

**Published:** 2022-05-28

**Authors:** Kathryn E. Luker, Gary D. Luker

**Affiliations:** 1 Department of Radiology, Center for Molecular Imaging, University of Michigan Medical School, 109 Zina Pitcher Place, A524 BSRB, Ann Arbor, MI 48109-2200, USA; kluker@umich.edu; 2 Department of Biomedical Engineering, University of Michigan Medical School, 109 Zina Pitcher Place, A524 BSRB, Ann Arbor, MI 48109-2200, USA; 3 Department of Microbiology and Immunology, University of Michigan Medical School, 109 Zina Pitcher Place, A524 BSRB, Ann Arbor, MI 48109-2200, USA

**Keywords:** chemokines, luciferase complementation, bioluminescence imaging, cancer metabolism

## Abstract

In response to CXCL12, CXCR4 and ACKR3 both recruit β-arrestin 2, regulating the assembly of interacting proteins that drive signaling and contribute to the functions of both receptors in cancer and multiple other diseases. A prior proteomics study revealed that β-arrestin 2 scaffolds pyruvate kinase M2 (PKM2), an enzyme implicated in shifting cells to glycolytic metabolism and poor prognosis in cancer. We hypothesized that CXCL12 signaling regulates PKM2 protein interactions, oligomerization, and glucose metabolism. We used luciferase complementation in cell-based assays and a tumor xenograft model of breast cancer in NSG mice to quantify how CXCR4 and ACKR3 change protein interactions in the β-arrestin-ERK-PKM2 pathway. We also used mass spectrometry to analyze the effects of CXCL12 on glucose metabolism. CXCL12 signaling through CXCR4 and ACKR3 stimulated protein interactions among β-arrestin 2, PKM2, ERK2, and each receptor, leading to the dissociation of PKM2 from β-arrestin 2. The activation of both receptors reduced the oligomerization of PKM2, reflecting a shift from tetramers to dimers or monomers with low enzymatic activity. Mass spectrometry with isotopically labeled glucose showed that CXCL12 signaling increased intermediate metabolites in glycolysis and the pentose phosphate pathway, with ACKR3 mediating greater effects. These data establish how CXCL12 signaling regulates PKM2 and reprograms cellular metabolism.

## 1. Introduction

Chemokine receptor CXCR4 and the atypical chemokine receptor ACKR3 (formerly CXCR7) regulate essential processes in normal physiology and numerous diseases, including cancer, atherosclerosis, neurodegeneration, and autoimmunity [1]. While both receptors share chemokine CXCL12 as a common ligand, each receptor shows distinct kinetics and signaling magnitude [2]. CXCR4 functions as a classic G-protein-coupled receptor with ligand-binding activating G proteins as part of signaling, while ACKR3 lacks the key amino acid motif associated with coupling to G proteins [3]. ACKR3 scavenges CXCL12 from the extracellular environment to shape the chemotactic gradients of this chemokine [4,5]. Both CXCR4 and ACKR3 share a common mechanism: the binding of CXCL12 triggers the recruitment of β-arrestin molecules (β-arrestin 1 or 2), with subsequent receptor internalization and the initiation of context-dependent β-arrestin signaling through ERK and other pathways [2,3].

A prior mass-spectrometry-based analysis found more than 100 different proteins that interacted with both β-arrestins. Additional proteins selectively interacted with β-arrestin 1 or 2 [6]. Preliminary studies showed the recruitment of some proteins and dissociation of others in response to signaling by selected G-protein-coupled receptors. In the context of cancer metabolism and tumor progression, pyruvate kinase M2 (PKM2) stands out as one particularly interesting binding partner for both β-arrestin 1 and 2. PKM2 is one isozyme of pyruvate kinases, enzymes that catalyze the final step in glycolysis. Unlike other isozymes of pyruvate kinase, PKM2 transitions between tetramers with high enzymatic activity and dimers with very low activity in glycolysis [7]. Tetrameric PKM2 promotes oxidative metabolism with the production of ATP in mitochondria. Dimeric PKM2, the oligomerization state that predominates in proliferating cancer cells, leads to aerobic glycolysis (also known as the Warburg effect) with the accumulation of upstream molecules in glycolysis. Cells with dimeric PKM2 also divert glucose metabolism to the pentose phosphate pathway, which counters oxidative stress and provides precursor molecules for the synthesis of nucleic acids [8]. Phosphorylation by ERK1 or ERK2 also drives PKM2 to dimers and monomers, the latter of which has been reported to translocate to the nucleus and upregulate genes driving cell-cycle progression and glycolysis [9]. Although the need for PKM2 in proliferating cancer cells remains uncertain, the increased expression of PKM2 commonly occurs in cancer and serves as a marker of poor prognosis in a wide range of malignancies [10,11].

Both CXCR4 and ACKR3 signaling pathways promote tumor growth and metastasis in breast cancer and multiple other malignancies, making these receptors potential targets for therapy and molecular imaging [12,13,14,15]. The tumor-promoting mechanisms reported for these receptors include the proliferation of malignant cells, angiogenesis, local invasion, homing of disseminated tumor cells to CXCL12-rich sites of metastasis, and establishment of immunosuppressive tumor environments. Metabolism reprogramming represents another hallmark of cancer [16], but few studies have investigated the potential effects of CXCL12 signaling through CXCR4 or ACKR3 on metabolic shifts in cancer cells.

Using cell-based and in vivo imaging combined with mass spectrometry analyses, we discovered that CXCL12 signaling through CXCR4 and ACKR3 regulates interactions between ERK2, β-arrestin 2, and PKM2. Both receptors reduce PKM2’s association with β-arrestin 2 and decrease the oligomerization of this enzyme, a characteristic shift in aerobic glycolysis. We also demonstrated that CXCR4 and, to a greater extent, ACKR3, promote the glucose metabolism through glycolysis and the pentose phosphate pathway, processes associated with the proliferation of cancer cells. These results advance the understanding of how CXCL12, CXCR4, and ACKR3 regulate intracellular signaling and highlight the functions of these pathways in shaping metabolism.

## 2. Materials and Methods

### 2.1. Cell Culture

We obtained MDA-MB-231 and 293T cells from the ATCC (Manassus, VA, USA) and verified the authenticity of cell lines by short tandem repeat profiling, performed using the University of Michigan Advanced Genomics Core. Previously, we described immortalized human mammary fibroblasts (gift of Daniel Hayes, MD, University of Michigan, Ann Arbor, MI, USA) stably expressing CXCL12-α fused to Gaussia luciferase or Gaussia luciferase only [17]. We maintained cells in DMEM (ThermoFisher Scientific, Waltham, MA, USA) with 10% fetal bovine serum, 1% glutamine, 1% penicillin/streptomycin, and Plasmocin prophylactic (InvivoGen, San Diego, CA, USA) in a 37° humidified incubator with 5% CO_2_. We renewed cultures from frozen stocks at least every three months. We generated spheroids of MDA-MB-231 cells and human mammary fibroblasts as described previously [18].

### 2.2. Chemicals and Chemokines

We purchased all recombinant chemokines and cytokines from R&D Systems (Minneapolis, MN, USA), chemicals from Tocris (Bristol, UK), and luciferin from Promega (Madison, WI, USA). We prepared reagents according to the manufacturer’s specifications.

### 2.3. Expression Constructs

We created all lentiviral constructs in pLVX EF1α (Takara Biosciences, San Jose, CA, USA) or FUW (gift of David Baltimore, California Institute of Technology, Pasadena, CA, USA). We fused to CBC in pLVX EF1alpha IRES mCherry, FUW IRES mTagBFP for CBGN, or FUW IRES mCitrine for CBRN. We appended luciferase fragments in frame with the coding sequence for the gene of interest, as described previously [2], ablating the stop codon and adding a Gly-Ser linker to the 3′ end of each ORFs. We initially tested complementation pairs consisting of fusions to N- and C-terminal fragments for each interacting protein and presented the results for pairs that produced the greatest change in complementation signal. We previously reported fusions of CXCR4 or ACKR3 to GFP [19,20]. To stably express pairs or triplets of interacting complementation proteins, we transduced MDA-MB-231 cells with a lentivirus of interest and then used flow cytometry to recover transduced cells based on the correct co-expressed fluorescent protein. For the transient expression of complementation reporters, we transfected 293T cells in 10 cm dishes with 5 µg of each interacting protein and/or receptor using calcium phosphate precipitation [12]. We plated cells for assays one day after transfection and began experiments two days after transfection.

### 2.4. Luciferase Complementation Assays

We performed assays as described previously by our group [2]. We seeded cells in 96-well black-walled plates in complete growth medium based on DMEM without phenol red. One day later, we changed medium to phenol red free DMEM with 1% serum. We equilibrated medium in a 37° humidified incubator with 5% CO_2_ prior to adding to cells, and then returned cells to the incubator for one hour before starting an assay. For experiments using specific chemical inhibitors, we added desired concentrations of an inhibitor to the cells before this one-hour time. We next added a small volume of luciferin to each well, waited 10 min before steady-state luminescence was reached, and then acquired a time 0 image prior to adding CXCL12 or another ligand. We placed cells in an IVIS Lumina instrument (Perkin Elmer, Waltham MA, USA) maintained at 37° and acquired images at the timepoints indicated in figure legends. We separated bioluminescence from click beetle green and red luciferases, as described previously [18]. We quantified bioluminescence as radiance. For some experiments, we presented data as the fold change values relative to control cells without added ligand.

### 2.5. Animal Studies

The University of Michigan Institutional Committee on Animal Use and Care approved all studies. We orthotopically implanted 5 × 10^5^ MDA-MB-231 breast cancer cells stably expressing CXCR4-GFP, ACKR3-GFP, or unfused GFP along with 5 × 10^5^ human mammary fibroblasts stably secreting CXCL12-α into bilateral 4th inguinal mammary fat pads of 8-10-week-old female NSG mice (n = 5 mice per group, n = 10 tumors per group) (Jackson Laboratory, Bar Harbor, ME, USA) as described previously [17]. We injected cells in 50-µL sterile 0.9% NaCl per mammary fat pad. Based on prior imaging studies, these group sizes provided 0.85 power to detect 25% differences in imaging signal with an α value of *p* < 0.05. Breast cancer cells also stably expressed the complementation pair of Arr2-CBGN and PKM2-CBC, as well as constitutively expressing FP650 to measure tumor burden. When tumors grew to a 3–4 mm diameter based on caliper measurements 18 days after implanting cells, we performed bioluminescence imaging for click beetle luciferase activity and fluorescence imaging for FP650, as detailed previously [12]. To quantify tumor growth using FP650 fluorescence, we normalized radiance for each tumor to the corresponding value for each mouse on day 16 and expressed data as fold change.

### 2.6. Western Blotting

We performed Western blots on total cell lysates, as described previously by our group, using antibodies to PKM2 phosphorylated at serine 37 and total PKM2 (ThermoFisher Scientific, Waltham, MA, USA) [21].

### 2.7. ^13^C_6_-Glucose Labeling

We seeded 1 × 10^6^ MDA-MB-231-CXCR4 or -ACKR3 cells in 6 cm dishes one day prior to the assay. After washing cells once with PBS, we incubated cells for 30 min at 37 °C in phenol red free DMEM with 0.2% Probumin BSA (Sigma-Aldrich, St. Louis, MO, USA). Following a wash with PBS, we added glucose-free, phenol red-free DMEM (ThermoFisher) with 25 mM ^13^C_6_-glucose (Sigma-Aldrich) and 0.2% Probumin BSA with 300 ng/mL CXCL12-α for 15 min at 37 °C (n = 3 per condition). To end the labeling, we rapidly aspirated medium, washed with 3-mL warm de-ionized water, filled the dish with liquid nitrogen, and placed the dish on dry ice before storing at −20 °C. The University of Michigan Metabolomics core analyzed samples by LC-MS and processed data as described previously [22].

### 2.8. Statistics

We performed all experiments at least twice, except for ^13^C_6_-glucose labeling and mass spectrometry analysis, which we conducted once. For cell-based experiments, the presented data represent mean values and standard error of the mean for quadruplicate samples. Mass spectrometry data represent mean values from triplicate samples. We prepared graphs and performed statistical comparison with GraphPad Prism software (Dotmatics, Boston, MA, USA. We analyzed data using Mann–Whitney tests, with *p* < 0.05 defining statistically significant differences.

## 3. Results

### 3.1. Differential Effects of CXCR4 and ACKR3 on Interactions with β-Arrestin 2

**Figure 1:**
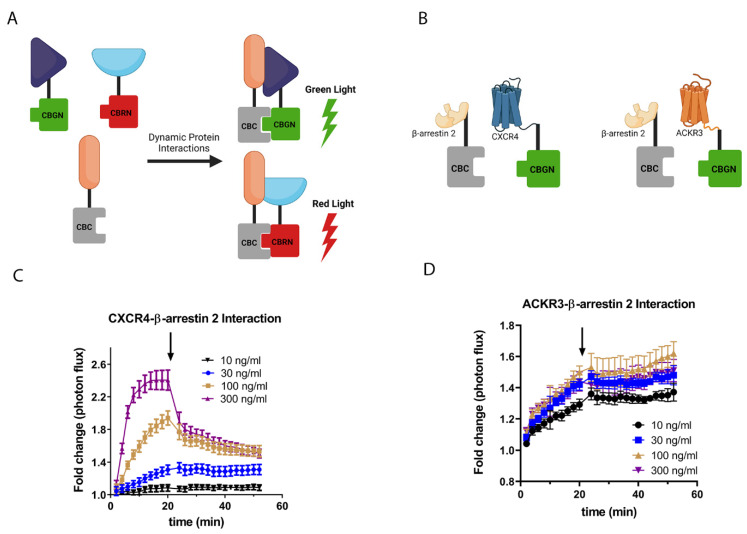
Differing kinetics of CXCL12-mediated recruitment and dissociation of β-arrestin 2 from CXCR4 and ACKR3. (**A**) Schematic of dual-color click beetle luciferase complementation system. We fused proteins of interest to N-terminal fragments of click beetle green (CBGN) or click beetle red (CBRN) luciferases or the common C-terminal fragment (CBC). Interactions between proteins of interest bring N- and C-terminal fragments together to produce light, with the N-terminal fragment determining the wavelength. We discriminated between green and red bioluminescence with optical filters. (**B**) Schematic of complementation pairs to detect association of CXCR4 or ACKR3 with β-arrestin 2 panels (**C**,**D**), respectively. (**C**) We treated MDA-MB-231 cells stably expressing CXCR4-CBGN (**C**) or ACKR3-CBGN (**D**) and β-arrestin 2-CBC with increasing concentrations of CXCL12-α. After 20 min of imaging, we washed cells to remove medium with CXCL12 (arrow) and then continued imaging during the washout phase. Graphs show mean values ± SEM for fold change in bioluminescence relative to cells treated with vehicle only. Illustrations created with BioRender.com (accessed on 28 April 2022).

Ligand binding to chemokine receptors and other seven transmembrane receptors triggers recruitment of the scaffolding protein β-arrestin 2, which promotes internalization of the receptor in endosomes. CXCL12 binding to CXCR4 stimulates receptor trafficking to lysosomes, where some of the internalized receptor is degraded to prevent prolonged signaling rather than being recycled to the cell membrane [23]. However, ACKR3 constantly internalizes and recycles to the cell membrane as part of the chemokine scavenging process [5]. We previously used luciferase complementation to measure the basal and CXCL12-dependent recruitment of β-arrestin 2 to CXCR4 or ACKR3 [2]. With the complementation system, the N-terminal fragment from either click beetle green or red luciferase determines the spectral emission of the complemented enzyme upon interaction with the common C-terminal fragment (CBC) (Figure 1A) [24]. The technology allows for the real-time quantification association and dissociation of interacting proteins fused to components of the complementation system. Our prior work showed that CXCL12-CXCR4 signaling drove a rapid, more transient recruitment of β-arrestin than ACKR3, which had higher basal levels of association and a slower, more prolonged interaction with β-arrestin 2.

Differences in receptor trafficking and recruitment kinetics led us to hypothesize that the association of ACKR3, but not CXCR4, with β-arrestin 2 would continue even after removing extracellular CXCL12. We used MDA-MB-231 cells that stably expressed CXCR4 or ACKR3 fused to the N-terminal fragment of click beetle green luciferase (CXCR4-CBGN or ACKR3-CBGN, respectively), and β-arrestin 2 fused to the CBC fragment (β-arrestin 2-CBC). We previously have shown these cells do not endogenously express either receptor in cell culture, allowing us to selectively analyze signaling by CXCR4 or ACKR3 [25]. We treated cells with increasing concentrations of CXCL12 to promote interactions between CXCR4 or ACKR3 and β-arrestin 2; then, we washed cells thoroughly to remove added chemokine before continuing with bioluminescence imaging (Figure 1B–D). Both receptors showed CXCL12-concentration-dependent recruitment of β-arrestin 2. The recruitment of β-arrestin 2 to ACKR3 was slower than that with CXCR4, at a comparable rate to our prior work. After removing CXCL12, the complementation signal for CXCR4-β-arrestin 2 promptly decreased, with the magnitude of decrease corresponding with the higher fold change in signal at the time of washing (Figure 1C). However, the complementation signal for ACKR3-β-arrestin 2 remained stable for almost 40 min after removing CXCL12, indicating the persistence of the ACKR3-β-arrestin 2 interaction in the absence of an extracellular ligand (Figure 1D).

**Figure 2:**
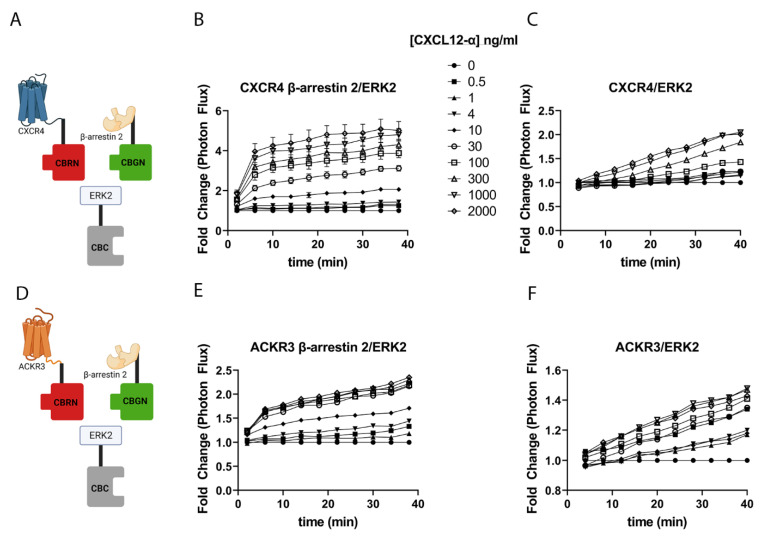
CXCL12 signaling through CXCR4 and ACKR3 promotes recruitment of ERK2 to β-arrestin 2 and receptors. (**A**,**D**) Schematic of dual-color click beetle luciferase complementation for association between CXCR4-CBRN (**A**) or ACKR3-CBRN (**D**) and β-arrestin 2-CBGN with ERK2-CBC. We used MDA-MB-231 cells stably expressing CXCR4-CBRN (**B,C**) or ACKR3-CBRN (**E,F**) with β-arrestin 2-CBGN and ERK2-CBC. We treated cells with increasing concentrations of CXCL12-α and imaged bioluminescence for 40 min, alternating images every 2 min in either green or red channels. The green complementation signal quantified the association of β-arrestin 2 with ERK2 (**B,E**), while the red channel measured interaction between the receptor and ERK2 (**C**,**F**). Graphs show mean values ± SEM for fold change in bioluminescence relative to cells treated with vehicle only. Error bars may be smaller than the presented symbol at each timepoint. Illustrations created with BioRender.com (accessed on 28 April 2022).

Following recruitment to a ligand-bound receptor, β-arrestin 2 may assemble and activate signaling molecules, such as ERK and other components of the MAPK pathway [26,27,28]. Relative functions of G proteins versus β-arrestin molecules in signaling show receptor- and cell-type-dependent differences. For example, CXCR4 signals through both G proteins and β-arrestin pathways, while ACKR3 largely functions as a β-arrestin-dependent, G-protein-independent receptor [3]. To investigate how CXCL12 signaling through CXCR4 versus ACKR3 controls formation of β-arrestin 2, ERK2, and receptor complexes, we generated cells with β-arrestin 2 fused to the N-terminal fragment of click beetle green luciferase (β-arrestin 2-CBGN); CXCR4 or ACKR3 fused to the N-terminal fragment of click beetle red luciferase (CXCR4-CBRN or ACKR3-CBRN), and ERK2 fused to the C-terminal fragment (ERK2-CBC) (Figure 2A,D). We stably expressed these complementation proteins in MDA-MB-231 cells, allowing for us to quantify the association between ERK2 and the activated receptor and β-arrestin. In cells expressing CXCR4, treatment with increasing concentrations of CXCL12-α triggered the association of β-arrestin 2 and ERK, which plateaued after approximately 24 min (Figure 2B). CXCL12 signaling through ACKR3 produced a different profile for the association between β-arrestin 2 and ERK2 (Figure 2E). Imaging showed a lower fold-change induction of bioluminescence than that measured with CXCR4. However, CXCL12-ACKR3 progressively generated an increase in the interaction between β-arrestin 2 and ERK2 over 40 min, similar to the progressive rise in complementation signal for the recruitment of β-arrestin 2 to ACKR3. We also measured the association between CXCR4 or ACKR3 and ERK2 using click beetle red bioluminescence. Both receptors showed progressively increasing interactions with ERK2 over time with a lower fold change than that measured for β-arrestin 2 and ERK2 in response to CXCL12 (Figure 2C,F).

**Figure 3:**
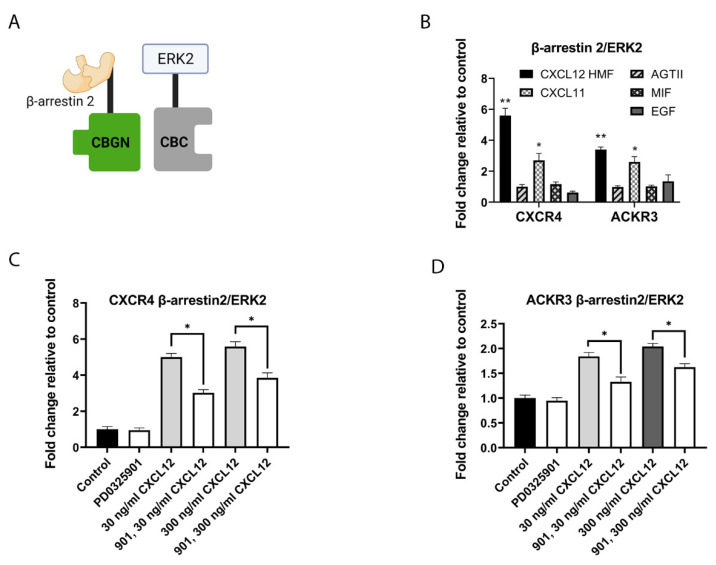
Modest effects of other ligands and compounds on interaction between β-arrestin 2 and ERK2. (**A**)Schematic of the complementation scheme for interaction between β-arrestin 2-CBGN and ERK2-CBC. (**B**) We treated MDA-MB-231 cells stably expressing CXCR4 or ACKR3 and the complementation pair of β-arrestin 2-CBGN and ERK2-CBC with 100 ng/mL CXCL12-α; 1 µg/mL CXCL11, angiotensin II, or MIF; or 100 ng/mL EGF. The graph shows mean values + SEM for bioluminescence normalized to control cells not treated with CXCL12-α measured 20 min after adding a ligand. *, *p* < 0.05 and **, *p* < 0.01 relative to control. (**C**,**D**) We treated MDA-MB-231 cells with CXCR4 (**B**) or ACKR3 (**C**) with 100 nM of the MEK inhibitor PD0325901 (901) for one hour before treating cells with listed concentrations of CXCL12-α. We quantified bioluminescence from interaction between β-arrestin 2-CBGN and ERK2-CBC 20 min after adding CXCL12. Graphs show mean values + SEM for fold change relative to control cells. * *p* < 0.05 and ** *p* < 0.01 for matched comparisons of the same concentrations of CXCL12 without or with PD0325901. Illustration created with BioRender.com (accessed on 28 April 2022).

To further investigate specificity for CXCR4 and ACKR3 signaling in the recruitment of ERK to β-arrestin 2 (Figure 3A), we tested other chemokine ligands reported to bind to one of these receptors (Figure 3B). As controls, we also treated cells with angiotensin II, a ligand for the angiotensin GPCR, or EGF, a ligand for receptor tyrosine kinase EGFR. MIF, previously described as a ligand for CXCR4 [29], did not increase interactions between β-arrestin 2 and ERK2 in cells expressing either CXCR4 or ACKR3. High (1 µg/mL) concentrations of CXCL11, another ligand for ACKR3 [30], enhanced the interactions between β-arrestin 2 and ERK2 in cells expressing either CXCR4 or ACKR3. Comparable increases in bioluminescence in cells with CXCR4 or ACKR3 likely occur because MDA-MB-231 cells endogenously express high levels of chemokine receptor CXCR3, which binds and signals in response to CXCL11 [31]. Neither angiotensin II nor EGF altered the association between β-arrestin 2 and ERK2 in MDA-MB-231 cells, although signaling through the endogenous angiotensin I receptor in MDA-MB-231 cells increased this interaction (Figure S1).

We also analyzed the effects of MEK, an ERK kinase in MAPK signaling, on interactions between β-arrestin 2 and ERK2. The phosphorylation of β-arrestin 2 by MEK has been reported to promote the recruitment and subsequent activation of ERK by GPCRs including CXCR4 [32]. For both MDA-MB-231-CXCR4 and ACKR3 cells, inhibiting MEK significantly reduced the CXCL12-dependent association between β-arrestin 2 and ERK (*p* < 0.05) (Figure 3C, D). We observed consistent effects at both tested concentrations of CXCL12, and PD0325901 alone did not significantly affect bioluminescence. These data demonstrate that MEK kinase activity is needed for the maximal recruitment of ERK2 to β-arrestin 2.

### 3.2. CXCR4 and ACKR3 Regulate Association of PKM2 with β-Arrestin 2

**Figure 4:**
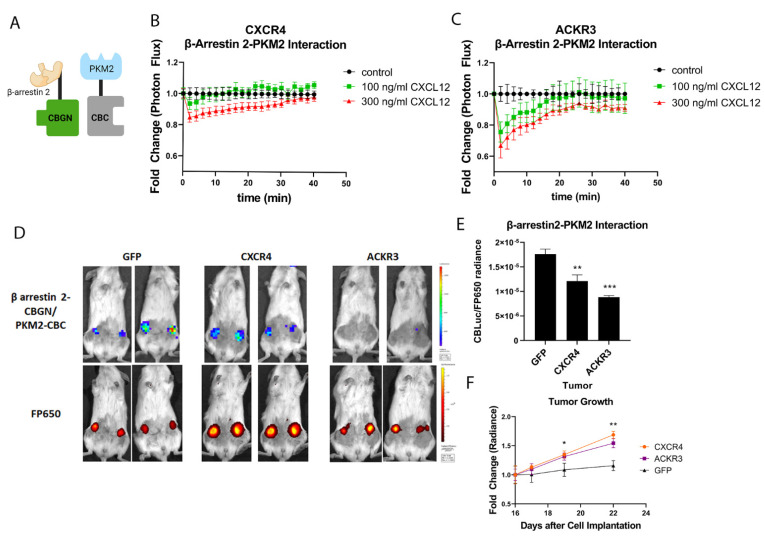
CXCL12 signaling leads to dissociation between PKM2 and β-arrestin 2. (**A**) Illustration shows complementation assay for association of β-arrestin 2-CBGN and PKM2-CBC. (**B**,**C**) We used cells stably expressing β-arrestin 2-CBGN and PKM2-CBC to quantify the interaction and dissociation between PKM2 and β-arrestin 2 in MDA-MB-231 cells expressing CXCR4 (**B**) or ACKR3 (**C**). We treated cells with listed concentrations of CXCL12-α and quantified bioluminescence over 40 min. Graphs show mean values ± SEM for basal association, and then CXCL12-stimulated dissociation of PKM2 from β-arrestin 2. * *p* < 0.05 and ** *p* < 0.01 relative to control cells not treated with CXCL12-α. (**D**) Representative images of mice with orthotopic tumor xenografts of MDA-MB-231 cells stably expressing β-arrestin 2-CBGN and PKM2-CBC and listed receptor or GFP control. We co-implanted human mammary fibroblasts secreting CXCL12-α. Images show pseudo-color displays of radiance from click beetle green complementation and fluorescence from FP650 stably expressed in cancer cells. (**E**) We normalized signal from click beetle green luciferase (CBLuc) to FP650 radiance in each tumor. ** *p* < 0.01 and *** *p* < 0.005 relative to GFP control tumors. N = 10 tumors per condition. (**F**) Graph shows growth in MDA-MB-231 tumors with CXCR4, ACKR3, or GFP based on fold change in FP650 radiance 14–22 days after implantation. * *p* < 0.05 and ** *p* < 0.01 relative to GFP control. Illustration in panel A created with BioRender.com (accessed on 28 April 2022).

Beyond the scaffolding components of the MAPK signaling pathway, a prior proteomics analysis identified that β-arrestin 1 and 2 bound the glycolytic enzyme, pyruvate kinase M2 (PKM2) [6]. The authors reported that signaling by the angiotensin I receptor qualitatively decreased the association between β-arrestin 2 and PKM2. However, this study did not investigate any other receptors or functional effects of the interaction between β-arrestin 2 and PKM2. To determine to what extent CXCL12 signaling regulates binding of β-arrestin- 2 to PKM2, we fused β-arrestin 2 to the CBGN fragment of click beetle green luciferase (Arr2-CBGN) and PKM2 to the common C-terminal fragment of click beetle luciferases (PKM2-CBC) (Figure 4A). We stably co-expressed β-arrestin 2-CBGN and PKM2-CBC in MDA-MB-231 cells stably transduced with CXCR4 or ACKR3 [20]. We treated cells with 100 or 300 ng/mL CXCL12-α and then measured changes in the interaction between Arr2-CBGN and PKM2-CBC by bioluminescence (Figure 4B,C). CXCL12 produced a concentration-dependent decrease in the association between β-arrestin 2 and PKM2, beginning with the earliest measured timepoint of 2 min. The interaction slowly recovered to baseline levels by 20–30 min. CXCL12 signaling through ACKR3 stimulated an increased dissociation of β-arrestin 2 and PKM2, with both 100 ng/mL and 300 ng/mL concentrations generating significant differences from vehicle one (*p* < 0.05 and *p* < 0.01 according to area-under-the-curve (AUC) analysis, respectively). By comparison, only the higher concentration of CXCL12 significantly reduced the complementation signal in cells with CXCR4.

To investigate the CXCL12-mediated regulation of the interaction between β-arrestin 2 and PKM2 in vivo, we used an orthotopic tumor xenograft model of human breast cancer. We implanted MDA-MB-231 cells stably expressing CXCR4, ACKR3, or GFP control and the complementation pair of β-arrestin 2-CBGN and PKM2-CBC into mammary fat pads of female NSG mice. Breast cancer cells constitutively expressed a far-red fluorescent protein, FP650, to normalize the luciferase complementation signal from Arr2-CBGN and PKM2-CBC to total numbers of cancer cells. We also implanted human mammary fibroblasts that secrete CXCL12-α, reproducing the secretion of this chemokine by carcinoma-associated fibroblasts in human breast cancers [33].

When mice developed 3–4-mm tumors, we imaged bioluminescence from Arr2-CBGN and PKM2-CBC and normalized the luciferase complementation signal to fluorescence from FP650. Relative to breast cancer cells expressing GFP control, the expression of CXCR4 and, to a greater extent, ACKR3, significantly reduced bioluminescence due to the association between β-arrestin 2 and PKM2 (*p* < 0.01 and *p* < 0.005 for CXCR4 and ACKR3, respectively) (Figure 4D,E). Although this study focused on quantifying interactions between Arr2-CBGN and PKM2-CBC, a limited analysis showed a greater growth in tumors with CXCR4 or ACKR3 based on increases in fluorescence from FP650 (Figure 4F). These data establish that CXCL12 signaling through receptors CXCR4 and ACKR promotes the dissociation of β-arrestin 2 from PKM2 in a breast tumor environment, pointing to a mechanism by which CXCL12 signaling pathways may regulate functions of PKM2 in cancer and promote the growth of breast tumors.

### 3.3. CXCL12 Signaling Reduces Oligomerization of PKM2

**Figure 5:**
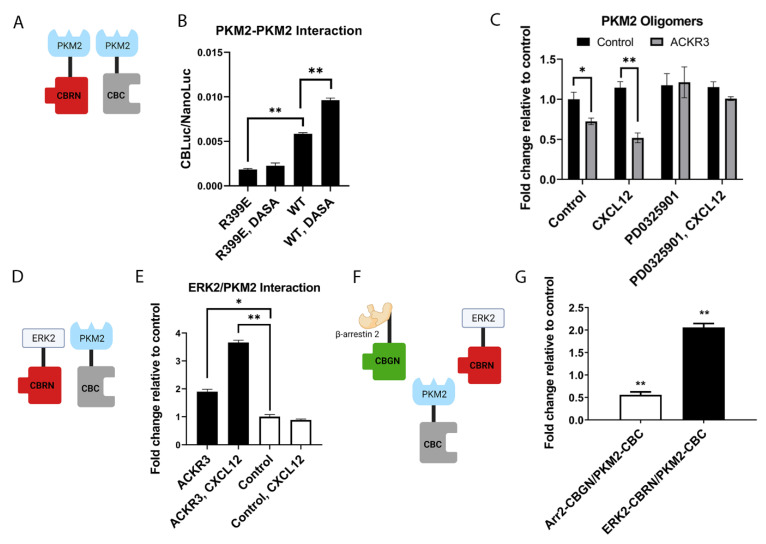
CXCL12-ACKR3 signaling reduces oligomerization of PKM2. (**A**) Illustration of click beetle complementation system for PKM2 dimers based on interaction between PKM2-CBRN and PKM2-CBC. (**B**) Graphs show mean values + SEM for bioluminescence produced by 293T cells transiently transfected with WT or R399E PKM2-CBRN and PKM2-CBC complementation pairs. We treated cells with 30 µM DASA-58, a compound that promotes tetramerization of WT PKM2, for 3 h before measuring bioluminescence. WT control cells and cells with R399E PKM2 received vehicle only. (**C**) We transiently transfected 293T cells with WT PKM2 oligomerization reporters and ACKR3-GFP or GFP control. We treated cells with 100 nM of the MEK inhibitor PD0325901 or vehicle for one hour before adding 100 ng/mL CXCL12-α. Graphs show mean values + SEM for bioluminescence measured 20 min after adding CXCL12 and normalized to control cells. (**D**) Complementation scheme for association between ERK2-CBRN and PKM2-CBC. (**E**) We transiently transfected 293T cells with WT PKM2 oligomerization reporters and ACKR3-GFP or GFP control. We treated cells with 100 nM of the MEK inhibitor PD0325901 or vehicle for one hour before adding 100 ng/mL CXCL12-α. Graphs show mean values + SEM for bioluminescence measured 20 min after adding CXCL12 and normalized to control cells. (**F**) Dual-color complementation assay for interactions of β-arrestin 2-CBGN or ERK2-CBRN with PKM2-CBC. (**G**) We co-transfected 293T cells with the complementation pair of ERK2-CBRN and PKM2-CBC and ACKR3-GFP or GFP control. Graphs depict mean values + SEM normalized to control cells for bioluminescence measured 20 min after adding 100 ng/mL CXCL12-α. (**D**) Graphs show mean values + SEM for bioluminescence in green and red channels from cells expressing β-arrestin 2-CGBN (Arr2-CBGN), ERK2-CBRN, and PKM2-CBC. We measured bioluminescence 20 min after adding 100 ng/mL CXCL12-α and normalized data to control cells not treated with CXCL12-α. * *p* < 0.05; ** *p* < 0.01. Illustrations created with BioRender.com (accessed on 28 April 2022).

PKM2 shifts between tetramers and dimers with high and low enzymatic activity, respectively, in glycolysis [34]. Tetrameric PKM2 promotes the pyruvate metabolism in mitochondria for oxidative phosphorylation in normal cells, while dimeric PKM2 in malignant cells favors the glycolysis and conversion of pyruvate to lactate. Since we previously used luciferase complementation to quantify changes in the oligomerization of receptors, we hypothesized that click beetle complementation would detect a relative abundance of higher- and lower-order oligomers of PKM2 (Figure 5A) [35]. As an initial test, we analyzed conditions that favor PKM2 tetramers or dimers. DASA-58 is a potent activator of PKM2, which shifts the enzyme to a tetrameric state, while the R399E mutation in PKM2 disrupts the tetramerization interface to favor dimers [36,37] We transiently transfected 293T cells with wild-type or R399E PKM2 fused to complementation fragments CBRN or CBC (PKM2-CBRN or PKM2-CBC, respectively). Cell-based imaging showed that the R399E mutation significantly reduced bioluminescence relative to cells with wild-type PKM2 (*p* < 0.01), while treatment with DASA-58 only increased the association of PKM2 in cells with the wild-type complementation pair (*p* < 0.01) (Figure 5B). Results from this experiment show that bioluminescence shifts as expected, with interventions that favor tetramers and dimers of PKM2, validating the complementation reporter system for relative changes in PKM2 oligomers.

We then used this reporter system to investigate the effects of CXCL12 signaling on PKM2, focusing on ACKR3 because this receptor had greater effects on the interaction between β-arrestin 2 and PKM2 in vivo. We transiently transfected 293T cells with ACKR3 fused to GFP or GFP control and the complementation reporter for PKM2 oligomers. We verified the comparable expression of ACKR3 and GFP based on fluorescence imaging (not shown). The expression of ACKR3 decreased the basal levels of PKM2 oligomers, likely due to the ligand-independent association of ACKR3 with β-arrestin 2 and activation of downstream signaling (*p* < 0.05) (Figure 5C). Treating cells with 100 ng/mL CXCL12-α further decreased bioluminescence, reflecting a shift from tetrameric to dimeric PKM2 (*p* < 0.01). MAPK signaling through ERK1/2 activates glycolysis, and phosphorylation by ERK2 shifts PKM2 to dimers [38,39,40] Based on these results and the arrestin-dependent activation of ERK1/2 by ACKR3 [3], we hypothesized that inhibiting ERK would at least partially reduce the effects of CXCL12-ACKR3 signaling on PKM2 oligomers. Treatment with the MEK inhibitor PD352901 inhibited the loss of bioluminescence caused by CXCL12 without affecting the baseline signal, indicating that CXCL12-ACKR3 signaling through MEK and ERK shifts PKM2 to dimers.

Based on these data, we investigated the association between ERK2 and PKM2. We transfected 293T cells with ACKR3-GFP or GFP control and the complementation pair of ERK2-CBRN and PKM2-CBC (Figure 5D). Even in the absence of CXCL12, the expression of ACKR3 increased the complementation signal from the association between ERK2 and PKM2, as compared with GFP control (*p* < 0.05) (Figure 5E). The treatment of cells expressing ACKR3 for 15 min with CXCL12-α further increased the association between ERK2 and PKM2 without altering bioluminescence in cells with GFP control. Mutations in MDA-MB-231 cells constitutively activate ERK, so treatment with CXCL12 did not produce detectable changes in those phosphorylated (active) using Western blot. However, CXCL12-ACKR3 signaling increased the phosphorylation of PKM2 at serine 37, a known phosphorylation site for ERK1/2 that promotes anerobic glycolysis (Figure S2) [39]. These data suggest that CXCL12-dependent localization and protein interactions for ERK provide a level of control when activating PKM2. To establish how CXCL12-ACKR3 signaling regulates the relationship between the interaction between PKM2 and β-arrestin 2 versus ERK2, we employed a pairwise complementation approach by transfecting cells with β-arrestin 2 fused to CBGN (β-arrestin 2-CGBN), ERK2-CBRN, and PKM2-CBC (Figure 5F). We also co-expressed ACKR3-GFP. Dual-color bioluminescence revealed that treatment with CXCL12-α reduced the interaction between PKM2 and β-arrestin 2 and increased complementation between PKM2 and ERK2 following treatment with 100 ng/mL CXCL12-α (Figure 5G), consistent with an inverse relationship between PKM2 protein interactions regulated by ACKR3 signaling.

**Figure 6:**
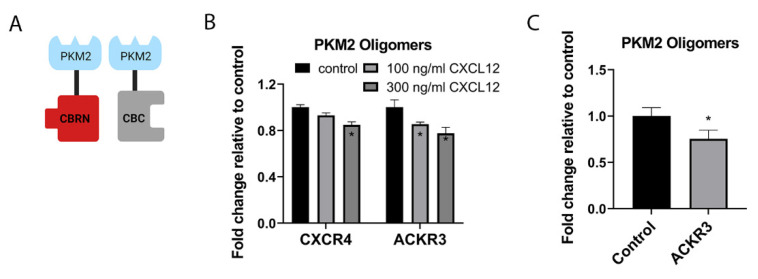
CXCR4 and ACKR3 signaling reduce PKM2 oligomers. (**A**) Schematic of click beetle complementation system for PKM2 dimers based on interaction between PKM2-CBRN and PKM2-CBC. (**B**) We used MDA-MB-231 cells stably expressing CXCR4 or ACKR3 and complementation pairs for oligomers of PKM2 (PKM2-CBRN and PKM2-CBC). We treated cells with listed concentrations of CXCL12-α for 20 min and quantified bioluminescence. Graphs depict mean values + SEM for bioluminescence normalized to control cells treated with vehicle only. (**C**) We formed spheroids of human mammary fibroblasts secreting CXCL12-α fused to Gaussia luciferase or unfused Gaussia luciferase and MDA-MB-231 cells with ACKR3 and the PKM2 oligomerization reporter. We measured bioluminescence after three days in culture. Graphs show mean values + SEM. * *p* < 0.05. Illustration created with BioRender.com (accessed on 28 April 2022).

We compared how CXCL12 signaling through CXCR4 versus ACKR3 regulates PKM2 oligomers. We stably expressed complementation reporters PKM2-CBRN/PKM2-CBC in MDA-MB-231 cells transduced with CXCR4 or ACKR3 (Figure 6A). We treated cells with increasing concentrations of CXCL12-α and quantified relative changes in bioluminescence for each interacting pair at 40 min. CXCL12 signaling produced concentration-dependent decreases in PKM2 oligomers for both CXCR4 and ACKR3, representing a more sustained shift to PKM2 dimers (Figure 6B). CXCL12 signaling through ACKR3 produced greater effects than CXCR4 regarding the dissociation of PKM2 oligomers, such as dissociation of PKM2 from β-arrestin 2 (see Figure 4A,B). To further investigate the effects of ACKR3 on PKM2 oligomers, we produced spheroids of MDA-MB-231 ACKR3 cells with human mammary fibroblasts stably expressing CXCL12-α fused to Gaussia luciferase or secreted Gaussia luciferase [41]. Due to the time required to form spheroids with this co-culture system and the slower diffusion of CXCL12 in spheroids relative to culture medium, we extended the incubation period to three days. Relative to control fibroblasts, spheroids with fibroblasts secreting CXCL12 significantly reduced bioluminescence from PKM2 oligomers (Figure 6C), demonstrating consistent effects of CXCL12-ACKR3 signaling in two- and three-dimensional environments.

### 3.4. CXCL12 Signaling Drives Aerobic Glycolysis and the Pentose Phosphate Pathway

**Figure 7:**
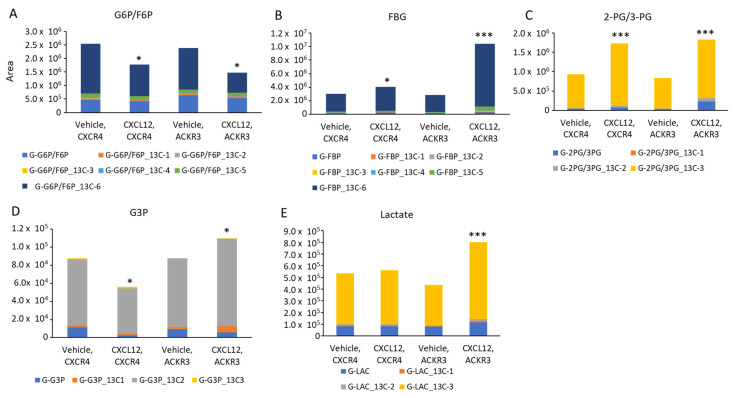
CXCL12 signaling through CXCR4 or ACKR3 regulates metabolites in glycolysis. We incubated MDA-MB-231 cells stably expressing CXCR4 or ACKR3 with ^13^C_6_ glucose and 300 ng/mL CXCL12-α or vehicle for 15 min and then harvested cells for mass spectrometry. Graphs show selected metabolites in glycolysis regulated by CXCR4 and/or ACKR3 signaling. For each graph, different colors show the metabolite and number of incorporated ^13^C molecules plotted as area from mass spectrometry analysis. Total height of each bar displays the sum of all detected molecules of the listed metabolite. (**A**) glucose-6-phosphate/fructose-6-phosphate; (**B**) fructose 1,6-bisphosphate; (**C**) 2-phosphoglycerate/3-phosphoglycerate; (**D**) glyceraldehyde-3-phosphate; (**E**) lactate. * *p* < 0.05; *** *p* < 0.005.

Luciferase complementation data showing the CXCL12-dependent dissociation of PKM2 oligomers suggested that CXCR4 and ACKR3 signaling shifted cancer cells toward glycolysis. To investigate the effects on glycolytic metabolism, we incubated MDA-MB-231-CXCR4 or ACKR3 cells with ^13^C_6_ glucose for 15 min before measuring labeled metabolites using mass spectrometry. As compared with vehicle control, cells treated with CXCL12 showed lower amounts of glucose-6-phosphate/fructose-6-phosphate and increased intermediates in glycolysis: fructose-1,6-bisphosphate (FBP) and 2-phosphoglycerate/3-phosphoglycerate (Figure 7A–E). We observed these changes in both the total amount of the metabolite and the subset with the highest incorporation of ^13^C. These data demonstrate an enhanced glycolytic metabolism of internalized glucose. For cells expressing ACKR3, stimulation with CXCL12 also increased glyceraldehyde-3-phosphate and lactate, the final product of glycolysis.

**Figure 8:**
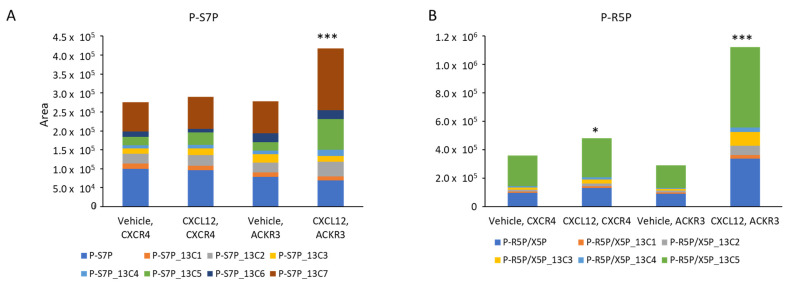
CXCL12 signaling through ACKR3 regulates metabolites in the oxidative and non-oxidative pentose phosphate pathway. We incubated MDA-MB-231 cells stably expressing CXCR4 or ACKR3 with ^13^C_6_ glucose and 300 ng/mL CXCL12-α or vehicle for 15 min and then harvested cells for mass spectrometry, as in Figure 7. Graphs show selected metabolites in the pentose phosphate pathway regulated by CXCR4 and/or ACKR3 signaling. For each graph, different colors show the metabolite and number of incorporated ^13^C molecules plotted as area from mass spectrometry analysis. Total height of each bar displays the sum of all detected molecules of the listed metabolite. (**A**) sedoheptulose-7-phosphate (non-oxidative pentose phosphate pathway); and (**B**) ribulose-5-phosphate (oxidative pentose phosphate pathway). * *p* < 0.05; *** *p* < 0.005.

In addition to glycolysis, the pentose phosphate pathway is another major catabolic pathway for glucose. Malignant cells in breast cancer and multiple other tumors commonly upregulate the pentose phosphate pathway to meet the metabolic demands of ongoing proliferation, including the production of nucleic acids and nicotinamide adenine dinucleotide phosphate (NADPH) to counter oxidative stress [42]. In cells expressing CXCR4, CXCL12 signaling through CXCR4 modestly increased the identified total and highest ^13^C-labeled metabolites in the pentose phosphate pathway: sedoheptulose-7-phosphate and ribulose-5-phosphate (Figure 8A,B). The treatment of ACKR3 cells with CXCL12 produced greater increases in these metabolites, particularly the subspecies with the highest incorporation of ^13^C from glucose. Overall, these data demonstrate that CXCL12 signaling promotes the glucose metabolism through two key pathways that drive tumor progression: glycolysis and the pentose phosphate pathway.

## 4. Discussion

**Figure 9:**
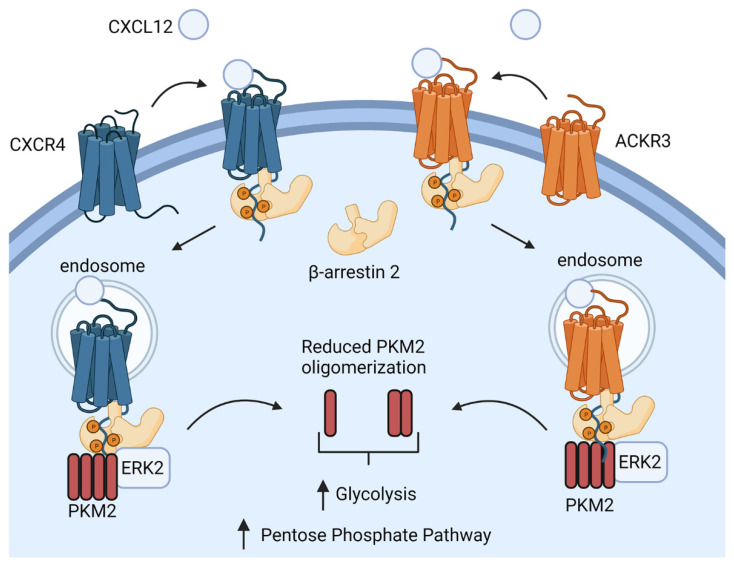
Summary of protein interactions from CXCL12 signaling to PKM2. CXCL12 binding to CXCR4 or ACKR3 triggers the recruitment of β-arrestin 2 and endocytosis of the receptor. ERK2 interacts with β-arrestin 2 and PKM2. PKM2 subsequently dissociates from β-arrestin 2 with PKM2, showing reduced oligomerization. We measure shifts from PKM2 tetramers to dimers and monomers from reduced complementation signal for PKM2 oligomers but do not directly quantify abundance of each species associated with or released from β-arrestin 2. We propose that dissociation from β-arrestin 2 and decreased oligomerization of PKM2 contribute to observed increases in metabolites in glycolysis and the pentose phosphate pathway, driven by CXCL12. Created with BioRender.com (accessed on 28 April 2022).

Building on our past work with luciferase complementation systems for imaging protein interactions in living cells, we established a unique suite of reporters to investigate intracellular signaling complexes regulated by CXCR4 and ACKR3. Figure 9 summarizes the observed protein interactions involving β-arrestin 2, ERK2, and PKM2 downstream of CXCL12 signaling through CXCR4 and ACKR3. CXCL12 triggers the recruitment of β-arrestin 2 to the activated receptor, a process previously shown to drive receptor endocytosis. β-arrestin 2 assembles multimeric protein interaction complexes involving ERK2’s associations with PKM2 and CXCR4/ACKR3, likely on endosomes based on prior studies of β-arrestin-dependent signaling [26,27,28]. This protein complex may facilitate the phosphorylation of PKM2 by ERK2, and the CXCL12-dependent dissociation of PKM2 from β-arrestin 2. CXCL12 signaling leads to the reduced oligomerization of PKM2 from enzymatically active tetramers to the dimers and monomers that promote glycolysis and glucose metabolism through the pentose phosphate pathway. Mass spectrometry analysis of ^13^C_6_ glucose revealed that CXCL12 signaling through CXCR4 and ACKR3 promotes the glucose metabolism through the pentose phosphate pathway and the accumulation of intermediate metabolites in glycolysis. While both CXCR4 and ACKR3 drive these protein interactions and metabolic shifts, we noted differences in the kinetics and magnitude of signaling responses and metabolites. CXCR4 triggered the greater induction and more rapid recruitment of β-arrestin 2 to the receptor, and the association between ERK2 and β-arrestin 2. By comparison, ACKR3 sustained interactions with β-arrestin 2 even after CXCL12 removal and produced greater changes in PKM2 oligomerization state and glucose metabolism. The causes of these differences in kinetics and outputs have yet to be fully defined. Nevertheless, this study establishes a previously unknown function of CXCL12 signaling to regulate PKM2 and glucose metabolism, connecting CXCR4 and ACKR3 to the metabolic adaptations of cancer cells.

Cells with a high demand for the synthesis of nucleic acids, particularly cancer cells, preferentially express PKM2 and maintain the enzyme as a dimer [43]. The low enzymatic activity of dimeric PKM2 results in the accumulation of upstream metabolites in glycolysis, which cells then can utilize to produce the necessary molecules for redox balance and proliferation. Dimeric PKM2 promotes aerobic glycolysis, also known as the Warburg effect, with glucose metabolized to lactate rather than acetyl CoA to support oxidative phosphorylation. We demonstrated that CXCL12 signaling releases PKM2 from β-arrestin 2 and reduces the oligomerization of PKM2 based on decreased bioluminescence from PKM2-PKM2 luciferase complementation. Based on prior work by our group using luciferase complementation to detect the oligomerization of proteins, we infer that reduced bioluminescence represents a shift from tetramers to dimers and possibly monomers [35]. However, our imaging technology measures relative changes in oligomerization and the lack of absolute amounts of tetramers, dimers, or monomers associated with β-arrestin 2 or released from this scaffolding protein. Our mass spectrometry analysis of glucose metabolism reveals the accumulation of intermediate metabolites in glycolysis and the pentose phosphate pathway in response to CXCL12 signaling through CXCR4 or ACKR3. Due to the mutations that constitutively activate ERK1 and ERK2 in MDA-MB-231 cells, we could not detect the CXCL12-dependent activation of these kinases downstream of CXCR4 or ACKR3. Numerous studies document that CXCR4 activates ERK1/2 [44]. The activation of ERK1/2 by CXCL12-ACKR3 occurs in some, but not all model systems, implying the context-dependent effects [45,46,47]. The correlation between the effects of dimeric PKM2 on glucose metabolism and our mass spectrometry data suggests that the regulation of interactions between β-arrestin 2 and PKM2 is the underlying mechanism of the metabolic effects of CXCR4 and ACKR3. CXCL12 likely promotes glycolytic metabolism through other processes activated by CXCL12 signaling through CXCR4 and/or ACKR3, including ERK and Akt. Collectively, these, and potentially other CXCL12-dependent pathways, support the increased proliferation of cancer cells previously reported by our group and others [12,13,48,49,50].

The scaffolding function of β-arrestin 2 potentially regulates other reported mechanisms of action for PKM2 in metabolism, as well as tumor progression. CXCL12 stimulates the recruitment of ERK2 to β-arrestin 2 and the formation of multi-protein interactions, including PKM2, which may facilitate the phosphorylation of PKM2, as detected downstream of CXCL12-ACKR3 signaling. The ERK phosphorylation of PKM2 is essential for aerobic glycolysis in cancer cells in cell-based assays and mouse models of cancer [39]. A prior study reported that PKM2 transferred via endosomes increases the expression of CXCL12 in recipient prostate cancer cells in bone marrow [51], which could initiate a feed-forward pathway, allowing for disseminated tumor cells to survive and proliferate. PKM2 may also enter the nucleus and regulate the expression of genes promoting proliferation and glycolysis [38]. Interaction with β-arrestin 2 under basal conditions may sequester PKM2 and prevent it from entering the nucleus until triggered by a driver of proliferation, such as CXCL12 signaling through CXCR4 or ACKR3. The scaffolding function of β-arrestin 2 controls the localization and functions of other interacting proteins. For example, ERK activated through β-arrestin only phosphorylates cytoplasmic targets, while ERK molecules activated by G proteins enter the nucleus to regulate gene expression [52].

The available evidence supports a strong correlation between the increased expression of PKM2 in cancer and the metabolic adaptations necessary for proliferation. However, ongoing studies also suggest complex, possibly context-dependent effects of PKM2 in cancer. Nuclear PKM2 has been reported as a protein kinase, phosphorylating histones and other epigenetic regulators to increase the expression of oncogenic proteins such as Myc- and cyclin-dependent kinases [38]. The protein kinase activity of PKM2 remains controversial. One study failed to detect the protein kinase activity of PKM2 and instead attributed this function to the contaminating molecules associated with PKM2 in biochemical enzyme preparations [53]. The necessity for PKM2 in the proliferation of cancer cells also remains unclear. Using a mouse model of breast cancer with the Cre recombinase-mediated deletion of PKM2, Israelsen et al. discovered that the loss of PKM2 at the time of tumor initiation accelerated tumor development [10]. Non-proliferating cells expressed PKM1, another isozyme of PKM that exists as an enzymatically active tetramer, while proliferating cells expressed minimal or no PKM isozymes. While demonstrating that tumor initiation does not require PKM2, this study and others support the hypothesis that the loss of enzymatically active PKM function, such as PKM1 downregulation, is essential for proliferating cancer cells [54,55]. Studies also suggest that the acute loss of PKM2, such as through Cre-mediated deletion, produces effects that differ from the chronic loss of this enzyme. These reports indicate that the effects of CXCL12 on PKM2 interaction with β-arrestin 2 and PKM2 oligomers in cancer and other diseases may vary based on cell type, environment, and time.

Overall, the current study reveals that CXCL12 signaling through CXCR4 or ACKR3 regulates PKM2 scaffolding on β-arrestin 2 and reduces the oligomerization of this glycolytic enzyme. Since dimeric PKM2 is known to drive the glycolytic metabolism of glucose and accumulation of metabolites in glycolysis and the pentose phosphate pathway, our signaling data provide one potential mechanism for CXCL12-stimulated shifts in the metabolism of breast cancer cells. We studied the signaling effects through only CXCR4 or ACKR3. However, we and others have shown that these receptors form hetero-oligomers with distinct signaling responses [56]. Future research will determine the extent to which the CXCL12-dependent regulation of PKM2 through CXCR4 or ACKR3, either alone or in combination, controls the metabolic states of cancer cells relative to other downstream effectors, with established effects on the metabolism. While aerobic glycolysis was formerly regarded as the hallmark metabolic feature of cancer cells, the evidence now supports metabolic plasticity as the critical adaptation that enables cancer cells to proliferate and survive therapy in different environments [57]. Ongoing work will investigate how CXCL12 signaling shapes overall metabolic plasticity in primary and metastatic tumors, including effects on the metabolism of other molecules, such as lipids. In summary, our work establishes that CXCL12 signaling controls PKM2, a central regulator of metabolism in proliferating cells, and highlights the effects of CXCR4 and ACKR3 on metabolic reprogramming in cancer.

## Data Availability

Data are available upon reasonable request.

## Supplementary Materials

The following supporting information can be downloaded at: https://www.mdpi.com/article/10.3390/cells11111775/s1. Figure S1: Angiotensin II signaling through the angiotensin I receptor drives the association between β-arrestin 2 and ERK2. Figure S2: CXCL12 signaling through ACKR3 increases the phosphorylation of PKM2 at serine 37.
