# Supplementary material for: The CXCL12/CXCR4/ACKR3 Signaling Axis Regulates PKM2 and Glycolysis
Source: Cells. 2022 May 28;11(11):1775. doi: 10.3390/cells11111775 (PMC9179862; doi:10.3390/cells11111775)
Supplement: Supplementary file 1 [file cells-11-01775-s001.zip › cells-1641520-supplementary.pdf]

Supplementary Materials

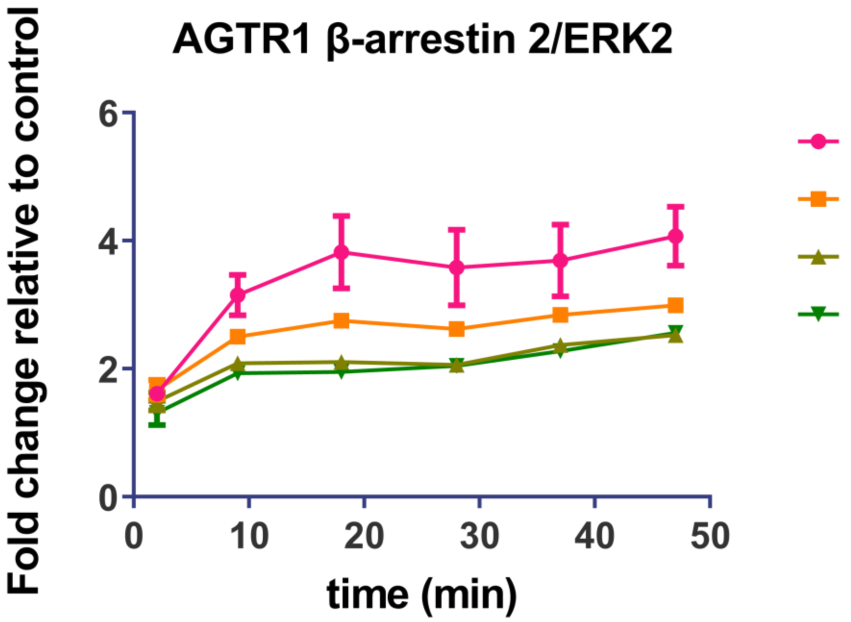

Figure S1. Angiotensin II promotes recruitment of ERK2 to β-arrestin 2. We used MDA-MB-231 cells expressing the angiotensin receptor 1 (AGTR1) and the complementation pair of for β-arrestin 2 and ERK2 (β-arrestin 2-CBGN and ERK2-CBC). We treated cells with listed concentrations of angiotensin II and quantified bioluminescence. Graph shows mean values ± SEM normalized to cell treated with vehicle only.

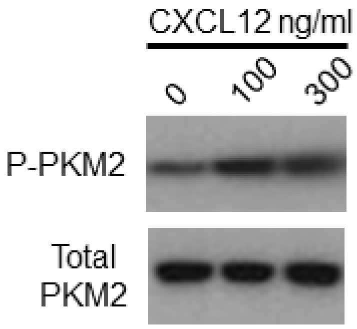

Figure S2. CXCL12-ACKR3 signaling increases phosphorylation of PKM2 at serine 37. We treated MDA-MB-231 cells expressing ACKR3 with 100 or 300 ng/ml CXCL12-α for 20 minutes and then harvested total cell lysates for Western blotting. Top row shows phospho-serine 37 PKM2. We stripped and re-probed the blot for total PKM2 (bottom row).
